# The withholding/withdrawing distinction in the end-of-life debate

**DOI:** 10.1186/2049-6958-9-13

**Published:** 2014-03-11

**Authors:** Virginia Sanchini, Cecilia Nardini, Giovanni Boniolo

**Affiliations:** 1 Dipartimento di Scienze della Salute, University of Milan, Milan, Italy; 2 Department of Experimental Oncology, Istituto Europeo di Oncologia, Milan, Italy

## 

End-of-life decisions are often associated with the perspective of the respiratory specialist. On one side, the physician may be confronted directly with these decisions regarding their own patients with an end-stage respiratory failure caused by a chronic conditions such as chronic obstructive pulmonary disease (COPD). On the other hand, several degenerative pathologies – such as degenerative neuromuscular conditions – require at some point the resort to the respiratory unit, oftentimes while the patient is still fully competent.

In 2007 Nava et al. [1] surveyed for the European Respiratory Society the situation of end-of-life decision-making in respiratory intermediate care units across Europe, and they found that “an end-of-life decision is taken for 21.5% of patients admitted” [1]. The number speaks for the relevance of the issue not only for critical care specialists but for all respiratory physicians. A further aspect to be considered is the unpredictable evolution of chronic respiratory conditions as compared to other diseases requiring end-of-life care. For tumour patients, it is generally possible to predict with accuracy the moment when a life-sustaining intervention will be needed. On the other hand, for respiratory ailments, it is difficult to predict the onset of a terminal respiratory failure. This potentially creates controversial situations where assisted ventilation is put in place to manage an acute event, but then has to be maintained and becomes a life-sustaining intervention in all respects. Managing situations like the one described is particularly complex for two main reasons. As a first thing, the fact that terminal stages of respiratory conditions have such blurred boundaries as opposed to other diseases such as cancer means that the physician may fail to recognise that he is dealing with a situation where an end-of-life decision is being taken. In case he does, though, the difficulty remains nonetheless, since we generally maintain that there is an ethically relevant distinction between “withholding” – i.e. not initiating – a life-sustaining intervention if the patient so wishes, and “withdrawing” the same treatment once it has been instated, even if the treatment is unwanted.

The distinction between “withholding” and “withdrawing” a life-sustaining medical treatment does, in fact, represent one of the most controversial issues in the end-of-life bioethical debate.

Before entering this debate, one should be mindful of the need to clarify the term “euthanasia”. Euthanasia generally means any action or omission that by itself or in its intentions leads to the death of the patient, in order to prevent his/her further suffering. This definition warrants further specification, i.e. that euthanasia is only a general expression to cover several different practices, further specified through the adjectives that are associated with this word: active and passive euthanasia; voluntary, non-voluntary and involuntary euthanasia; direct and indirect euthanasia.

The categories of withholding and withdrawing a treatment deal with the ones of active and passive euthanasia. Indeed, most bioethicists appear willing to define withdrawing a treatment as a form of “active” euthanasia (to perform an act that by itself causes the death of the patient), and withholding a treatment as a form of “passive” euthanasia (not to administer a lifesaving medical treatment, as a consequence of which the patient dies)^a^. As far as the ethical point of view is concerned, the relevant question does not really deal with the investigation of the two actions *per se*, but with the question of whether withholding and withdrawing a treatment are morally equivalent due to the same effect they cause. Providing an answer to this question means first and foremost addressing another question, that is whether “action” (in this case interrupting a process already started) and “omission” (in this case refraining from starting a process) might be considered equivalent from a moral point of view [2]. In order to remove any doubt, it would be useful to point out that this question is not concerned with definitively establishing whether it is ethically allowed to withhold and/or withdraw a lifesaving medical treatment to a dying patient. Instead, it establishes that if withholding and withdrawing (hence if action and omission) are morally equivalent, then either both of them are ethically allowed or both of them are ethically disallowed. The different ways in which bioethicists have answered this central question are only tangentially related to the better-known threads of the debate about euthanasia and assisted suicide (generally expressed as follows: “sanctity” of life vs. “quality” of life).

The traditional answer to this issue might be presented through a statement declared by the House of Delegates of the American Medical Association on December 4, 1973:

*“The intentional termination of the life of one human being by another – mercy killing – is contrary to that for which the medical profession stands and is contrary to the policy of the American Medical Association. The cessation of the employment of extraordinary means to prolong the life of the body when there is irrefutable evidence that biological death is imminent is the decision of the patient and/or his immediate family. The advice and judgment of the physician should be freely available to the patient and/or his immediate family”*.

According to this statement, therefore, while active euthanasia should be condemned both legally and morally, passive euthanasia might be, under some precise and circumstantial conditions, accepted. The main reasons lying behind such a choice seem to deal with the concept of “causality”. In the active euthanasia case, the physician is directly causing the patient’s death, whereas in the passive euthanasia case it is the terminal disease that eventually leads to the death of the patients. Could the same reasoning hold in the case of the withholding/withdrawing distinction as well? For instance, it might be argued that in the withdrawing scenario the physician interrupting the treatment is the direct cause of the patient’s death, whereas in the withholding case the physician does nothing to causally contribute to it.

That the causality argument is not a decisive reason to support a distinction also at the moral level between withholding and withdrawing a lifesaving medical treatment is what has been argued by some upstream bioethicists. James Rachels, for example, explores the ethical boundaries of the distinctions by means of a mental experiment now famous in bioethical literature. Rachels imagines two different scenarios: “In the first, Smith stands to gain a large inheritance if anything should happen to his six-year-old cousin. One evening while the child is taking his bath, Smith sneaks into the bathroom and drowns the child, and then arranges things so that it will look like an accident. In the second, Jones also stands to gain if anything should happen to his six-year-old cousin. Like Smith, Jones sneaks in planning to drown the child. However, just as he enters the bathroom Jones sees the child slip and hit his head, and fall face down in the water. Jones is delighted; he stands by, ready to push the child’s head back under if it is necessary, but it is not necessary. With only a little thrashing about, the child drowns all by himself, “accidentally,” as Jones watches and does nothing” [3]. Rachels claims that when we examine two cases in which the results are the same and the motives (personal gains) are the same, the simple reason that there is a “factual” difference between killing and letting die does not provide any robust justification in favour of a “moral” difference between the two.

It is clear from the foregoing discussion that the distinction between withholding and withdrawing of a life-sustaining treatment is far more complex than a simple dichotomy, and that its ethical ramification has profound consequences for end-of-life decisions in the clinical practice. We trust therefore that the readers of *Multidisciplinary Respiratory Medicine* (MRM) will be interested in the series of papers that a selected group of authors (Jos Weile, Henk ten Have, Massimo Reichlin, Michael Barilain e Patrizia Borsellino) have contributed to this Journal in a comprehensive exploration of the state-of-the-art on this ethical issue.

The contribution of Jos Weile and Henk ten Have *The Ethics of Forgoing Life-Sustaining Treatment – Theoretical Considerations* might be considered a first step in this direction. They provocatively argue that the default option in the end-of-life context does not rest in curing the patient but in following almost always the imperative of “do not treat”. Indeed, two main conditions should be met, according to them, before physicians and health care professionals in general are morally permitted to provide a treatment. Firstly, the treatment must be medically indicated, that is such a treatment “is likely to benefit the patient and not cause disproportionate harm”. Secondly, the patient “must be informed about his diagnosis, prognosis, and the nature of the treatment X, and must consent to it”. Hence, following their reasoning, not only action and omission have two different moral weights, but omitting a treatment is nearly always a better option from an ethical point of view than providing the patient with a treatment, because the administration of any treatment is bound to the fulfillment of the above-mentioned conditions.

The belief that it is not always necessary to impose on the patient lifesaving medical treatments constitutes a shared idea among the contributors to this debate in our Journal. In addition to the paper presented in this issue of MRM, three more articles on the debate are going to appear in a subsequent issue. The authors analyse the withholding-withdrawing distinction from different perspectives and areas of expertise, but they all share the same underlying ethical belief of “not imposing on the patient unwanted treatments”. Massimo Reichlin in *On the ethics of withholding and withdrawing medical treatment* investigates precisely this concept. After having argued in favour of the moral irrelevance of the distinction between withholding and withdrawing and of the ethical legitimacy of the patient’s choice over both these options, he clarifies that this idea does not necessarily imply the ethical legitimacy of a form of voluntary active euthanasia. Indeed, whether the latter is bound to the acceptance of a robust positive right, ascribable to the patient, to decide when to terminate their life (which should confer, at the same time, a right to the physician to kill this patient), the rationale underlying withholding or withdrawing a treatment is completely different. Through the author’s words, it is “a moral principle according to which the patient has a right to decide the therapies he is willing to accept and those he does not want”. Rephrased in this way, the debate should be rethought in the light of two considerations. On the one hand, we might argue that it is the conditions in which action and omission take place that makes a moral difference rather than action and omission as such. On the other hand, also omission should be rethought, since “What justifies this decision is not the physician’s doing nothing, but the fact that the patient and the physician are agreed that the benefits to be gained by insisting on the treatment do not justify the burdens that it imposes on the patient, considering his situation and quality of life”.

The last two papers distance themselves from the perspective considered so far. Despite maintaining the same ethical imperative of not imposing on the patient unwanted and potentially harmful treatments, they address such a topic either from a sociological or from a legal perspective.

In *Rethinking the withholding/withdrawing distinction: the cultural construction of “life support” and framing of the end-of-life decisions*, Y.M. Barilan, through the case of the Israeli 2005 Law on patient nearing death and its latest developments, shows how traditional distinctions framing bioethical debate over the end-of-life focus on technical aspects of human action and that these distinctions might be completely reversed by introducing some devices to, for examples, machines for artificial ventilation. Because of that, this article interestingly proposes to reframe the classical bioethical distinction at stake into broader historical, psychological and regulatory context. In particular, through the analysis of two hypothetical case scenarios and the above-mentioned Israeli law, Barilan shows how the law exercises its regulatory power far beyond the cases it is supposed to regulate, thus suggesting that technology and social values are thoroughly entwined.

Finally, in *Limitation of the therapeutic effort: ethical and legal justification for withholding and/or withdrawing life sustaining treatments*, Patrizia Borsellino identifies withholding and withdrawing as two forms of limitation of the therapeutic efforts and upstream argues that there are criteria “both in medical ethics in the law often adequate enough to remove doubts, and to guide decisions and actions”. Indeed, first of all, she claims that provided that we are in the case of a competent patient, “the current legal-ethical frameworks subordinate all medical interventions to the patient’s will”. Instead, in the case of patient lacking decision capacity, most European and non-European countries envisage the appeal to the so-called living will or the reference to a substitute person to make their health care decisions and to participate in the decision-making process.

Assistance to end-of-life decision-making is, and will probably remain, a troublesome area for the respiratory physician, also due to the fact that the literature is most often dealing with the situation of acute patients admitted to the respiratory unit, rather than of chronic respiratory patients facing end-stage respiratory failure. We believe that exploring the distinction between withholding and withdrawing life-sustaining treatment represents a valuable effort towards bringing the ethical discourse closer to the practice of the physician in general and of the pulmonary specialist in particular. The series of papers we collected address this topic from different perspectives (ethical, legal, sociological) and, by thoroughly investigating this distinction, they are able to clarify a debate in which the boundaries between “the rationally consistent” and “the personal arbitrariness” are not always so easy to be established.

## Endnotes

^a^Actually, some bioethicists would disagree with such an analogy. Indeed, some philosophers think that by passive euthanasia we should consider the interruption of a medical treatment still useful for patient’s life, whereas in the case of both withholding and withdrawing we deal with a medical treatment useless and disproportionate for the patient’s life.
